# Ovarian and Renal Vein Thrombosis: A Rare Cause of Fever Outer the Postpartum Period

**DOI:** 10.1155/2015/817862

**Published:** 2015-06-22

**Authors:** Turhan Togan, Hale Turan, Egemen Cifci, Ceylan Çiftci

**Affiliations:** ^1^Department of Infectious Diseases and Clinical Microbiology, Başkent Universitesi, Konya Uygulama ve Arastirma Merkezi, Hoca Cihan Mahallesi Saray Caddesi, No. 1, Selcuklu, Konya, Turkey; ^2^Department of Radyoloji, Başkent Universitesi, Konya Uygulama ve Arastirma Merkezi, Hoca Cihan Mahallesi Saray Caddesi, No. 1, Selcuklu, Konya, Turkey; ^3^Department of Obstetrics and Jınekoloji, Başkent Universitesi, Konya Uygulama ve Arastirma Merkezi, Hoca Cihan Mahallesi Saray Caddesi, No. 1, Selcuklu, Konya, Turkey

## Abstract

Although there is no other underlying disease, women can sometimes experience rare and serious diseases such as ovarian vein thrombosis (OVT) and renal vein thrombosis (RVT) after giving birth. The widespread development of thrombosis is treated for the first time in this study. Stasis, coagulation factor abnormalities, and intimal damage to the venous thrombosis risk can increase during pregnancy. It was mentioned that it diagnoses an abnormality in the hypercoagulability half of women with OVT. Despite the hypercoagulant abnormality observed in pregnant women, it was very unusual that the renal vein thrombosis led to this complication. It can lead to severe complication of OVT which can even cause death. It was the first time that the renal vein and ovarian vein thrombosis were observed in the postpartum period, and there was no coagulation abnormality. It is known that the thrombus in the postpartum period can be observed with the fever of unknown origin. The problematic, but rarely observed, postpartum disease such as ovarian venous thrombosis (OVT) is generally observed in the right ovarian vein. In this disease, avoiding the resulting laparotomy heparin and intravenous antibiotics is best solution for the patient. If it is to be noted a fever for unknown reasons, that it be thrombosis.

## 1. Background

Postpartum women can sometimes experience rare and severe diseases such as ovarian vein thrombosis (OVT) and renal vein thrombosis (RVT) which may occur due to the pelvic inflammatory disease and surgical procedures as well as malignancies. The doctor needs to be careful to diagnose these rare diseases which emerge with abdominal pain complaints since the symptoms are similar to the acute abdomen. The ovarian and renal vein thrombosis on the third day of the postpartum period were detected in a 29-year-old patient who was admitted to the hospital with a fever of unknown origin. Even though there was no other underlying disease, the widespread development of the thrombosis has been discussed for the first time in this study.

## 2. Case Report

The female patient (29 years old) with the complaints of high fever, chill, and shivering was admitted to the outpatient clinic of the infectious diseases. The patient had given birth to her second child 11 days before she was hospitalized. She used amoxicillin-clavulanic acid due to her fever emerging on the 3rd day of the postpartum period. She was admitted to our clinic because of her high fever despite the antibiotherapy. Blood and urine cultures were obtained from the patient for the preliminary diagnosis of pelvic inflammatory disease and then she was given ceftriaxone and metronidazole. She was not feeling healthy. The patient gave birth to a male baby with a natural vaginal delivery and there was no complication in the postpartum period. Additionally, she had the negative clinical history. On the other hand, she had the hypercoagulation status and cancer in her family history.

She did not have nausea and vomiting as well as vaginal bleeding. According to her physical examination and system queries, there was no finding regarding the infectious diseases. There was no pathological finding in her obstetric-gynecologic examination. The signs of the patient were as follows: orally measured body temperature was 38.7°C, blood pressure was 140/94 mm Hg, the pulse was 108 beats/min, the respiratory rate was 22 breaths/min, and the oxygen saturation was 100%. The hematological parameters of the patients with ovarian and renal vein thrombosis: APTT: 33,5 seconds, PTZ-INR: 1,09, PTZ: 13,3 seconds, bleeding time: 1 minute, platelet count: 175100 K/mm^3^, antithrombin III: 109%, protein S antigen: 62%, protein C antigen: 90%, and fibrinogen: 503 mg/dL.

**Figure 1:**
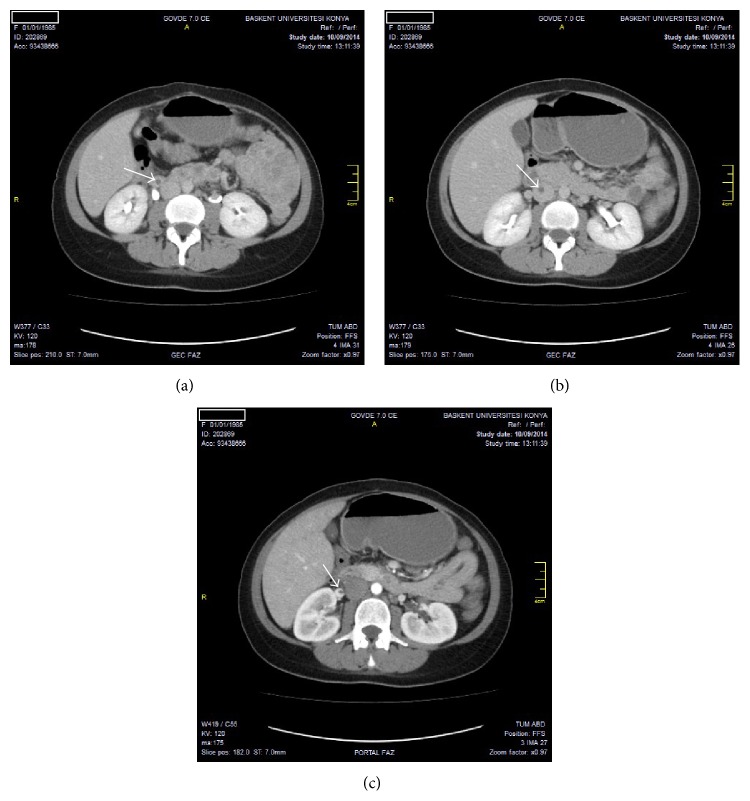
(a) Ovarian vein thromboses. On axial CT image, hypodense thrombus seen in right ovarian vein. (b) Inferior vena cava vein thromboses. On axial CT image, hypodense thrombus seen in vena cava inferior. (c) Renal vein thrombosis. On axial CT image, hypodense thrombus seen in right renal vein.

According to laboratory tests, the number of leukocytes was 8370 mm^3^, hs-CRP was 236 mg/L, and sedimentation rate was 69 mm/h. Biochemical parameters were within normal limits. Urine analysis was normal and there were no microorganisms in both blood and urine cultures. According to the molecular genetic analysis, MTHFR C677T and Factor V Leiden 1691 D>A results were evaluated as normal. The results of the homocysteine level analysis, Russell venom time (dRVVT), lupus anticoagulant, and anticardiolipin antibody were found to be negative. Chest X-ray was observed to be normal. There was a heterogeneity in the myometrium and a fluid in the endometrial cavity according to her abdominal USG. There was an appearance consistent with edema in her right over periphery according to the abdomen CT and thrombus in the right ovary vein, the right renal vein, and the inferior vena cava (Figures 1(a), 1(b), and 1(c)). There was no deep vein thrombosis but the patent venous system was observed in the bilateral lower extremity venous Doppler imaging. Echocardiogram findings were normal.

The anticoagulant treatment (enoxaparin 2 × 60 mg, subcutaneous) was started and she had the normal temperature on the 4th day. She was discharged from the hospital after the 7th day due to her good health status. She received the anticoagulation therapy with warfarin (INR 2-3) orally and she did not have the symptoms in the following 6 months after being discharged. There was no other complication during the 6-month follow-up.

## 3. Discussion

It has been reported that women can experience the postpartum ovarian thrombophlebitis (POVT) during their pregnancy (1/2000 to 1/600) or caesarean (1/800) [1]. Venous stasis is increased due to the increment in the ovarian venous system that can be almost three times enlarged during the pregnancy [2]. It has been stated that the 0,05 and 0,18% of the 30- to 40-year-old pregnant women are affected by OVT. Generally, the OVT is observed in the right ovarian vein after 2 to 15 days of the birth in 90% of the cases. The risk of the thrombosis is increased 1-2% with the caesarean and generally the risk factor for this complication is multiparity [2, 3]. Although women have cancer that is controlled with chemotherapy, it is the risk factor for the OVT development; frequently the asymptomatic thrombus is recovered in the absence of any treatment [4]. OVT can start to be developed due to the bacterial damage in the ovarian venous endothelium [5]. Bacterial entry is facilitated due to the interaction between the uterine, vaginal, and ovarian venous plexuses [5, 6]. The sepsis and the pulmonary embolism are the important complications of the OVT. Systemic lupus erythematosus, antiphospholipid syndrome, factor V Leiden presence, paroxysmal nocturnal haemoglobinuria, hyperhomocysteinemia, deficiency of the proteins C and S, and thrombocytopenia induced by heparin are the hypercoagulation diseases that are known as the risk factors for OVT [7, 8].

Postpartum OVT often develop (90%) in the right ovarian vein. There can be three reasons for this. For instance, compressing the right ovarian vein due to the dextroversion of the uterus can be one of these reasons. Furthermore, the antegrade flow in the right ovarian vein or the relatively more incompetent valves of the right ovarian vein can also lead to this disease. Nevertheless, the right ovarian vein is frequently associated with OVT in pregnancy [9].

These two diseases named as ovarian vein thrombosis and septic pelvic thrombophlebitis are associated with Virchow's triad of vessel wall injury, venous stasis, and hypercoagulability. There is an increased risk for thrombosis in women during the pregnancy and upon the delivery. The risk of this thromboembolic disease increases 5 times in the presence of pregnancy. There are pregnancy dependent reasons for the development of the thrombosis in the deep veins of the lower limbs and pelvis. One of these reasons can be the pressure on the inferior vena cava due to the uterus. Besides, the hormonal changes can also lead to this complication. Nevertheless, the coagulation and fibrinolysis alterations are thought to be the most important factors for the development of these diseases. Blood flow speed is rapidly slowed down upon giving birth. According to the results of Simons et al., there is blood flow in opposite directions in ovarian veins (anterograde flow in the right and retrograde flow in the left ovarian vein) that can rapidly occur after the birth. Besides, it has been indicated that there is a size difference between the right and the left ovarian veins and the longer right ovarian vein was shown to carry many valves. It has further been stated that these valves can be the risk factor for the thrombosis formation [10, 11]. Consistent with the literature, we observed the thrombosis in the right ovarian vein in our patient.

There are some signs such as pelvic pain, fever, and a right-sided abdominal mass that are observed in the first week after the delivery [1]. Renal vein thrombosis can develop without any specific sign and single lower limb edema can be the nonspecific symptom for this complication. It can also give acute signs such as flank pain, gross hematuria, anemia, fever, and proteinuria. The renal failure can happen in a short time if the bilateral thrombosis develops in a single kidney [12]. Even though the hypercoagulant abnormality is observed in pregnant women, it is very uncommon that renal vein thrombosis leads to this complication. There can be severe complications of the OVT which can even lead to death. These can be sepsis, extension of the thrombus to the inferior vena cava and renal veins, and pulmonary embolism. The pulmonary embolism (the incidence is 13.2%) is the most frequent reason for the morbidity due to OVT (as high as 5%). OVT can be mostly followed by sepsis and pulmonary embolism. The percentage of the pulmonary embolism observed in OVT can be 13%. It has been mentioned that there is a hypercoagulation abnormality in 50% of the women diagnosed with OVT [13]. Furthermore, if this hypercoagulation abnormality is not treated, the following pregnancy is not suggested for the prophylaxis.

Ultrasound, CT scan, and MRI observations are the techniques that can be used to diagnose the OVT. The sensitivities of CT scan and Doppler ultrasonography are >95% and ~50%, respectively [9]. However, the most sensitive and specific diagnosis can be achieved by using magnetic resonance angiography. On the other hand, the magnetic resonance angiography is used when there is a doubt and the MRI, ultrasound or MRI are frequently used because it is cheap to perform and fast to obtain the results [14].

In the postpartum period, the OVT treatment regularly can be performed by giving anticoagulants and antibiotics intravenously. The oral anticoagulant therapy is suggested for 3–6 months and the oral antibiotic treatment is recommended generally for 1 week unless the patient is in the hospital. Nevertheless, the length of the anticoagulant or antibiotic treatments has not yet been standardized. There can be some alternative ways to heal the patients in case the regular medication cannot stop the complication or if there is another serious abnormality. One of these alternative options can be the placement of an IVC Greenfield filter to hysterectomy and thrombectomy. Besides, the ligation of the inferior vena cava can even be performed [15].

It is the first time that renal vein and ovarian vein thromboses are observed in the postpartum period and even there is no coagulation abnormality. According to the current case, common thrombus extending from inferior vena cava to ovarian and renal vein in the postpartum period can be detected even without an additional underlying risk factor. It should be known that the thrombus formation in the postpartum period can lead to the fever with unknown reason.

## 4. Conclusion

It has been known that the severe but rarely observed ovarian vein thrombosis (OVT) is a postpartum disease and it is generally observed in the right ovarian vein. Even though color Doppler ultrasonography and magnetic resonance imaging can give a hint about the disease, CT scanning can be used for the exact diagnosis. In this disease, it is favourable to give the heparin and intravenous antibiotics to the patient in order to bypass the laparotomy. When there is a fever with unknown reason, it should be noted that it may be the thrombosis.
